# Tunable multichannel Fibonacci one-dimensional terahertz photonic crystal filter

**DOI:** 10.1038/s41598-023-32769-0

**Published:** 2023-04-06

**Authors:** V. Sepahvandi, B. Rezaei, A. H. Aly

**Affiliations:** 1 Faculty of Physics, University of Tabriz, Tabriz, Iran; 2 TH-PPM Group, Physics Department, Faculty of Sciences, Beni-Suef University, Beni Suef, 62521 Egypt

**Keywords:** Applied optics, Optical materials and structures

## Abstract

This paper proposes a multichannel terahertz optical filter based on a one-dimensional photonic crystal with a third-order Fibonacci structure, including a bulk Dirac semimetal. The tuning of the optical properties of the proposed structure has been theoretically studied as a function of the Dirac semimetals' Fermi energy. Furthermore, the effects of the Fibonacci structure's periodic number and light's incident angle on optical channels were investigated. The results reveal that changes in the Fermi energy and incident angle remarkably affect the frequency and transmission of the optical channels. Additionally, the number of optical channels increases by increasing the periodic number of the Fibonacci structure.

## Introduction

Photonic crystals (PhCs), also known as photonic band gap (PBG) materials, are artificial periodic materials predicted by Yablonovitch^1^ and John^2^ in 1987. These materials are periodic in one, two, or three directions and are designed to control light in a way^3–5^, which makes it feasible to develop new optoelectronic devices unattainable with conventional optics^6^. The main feature of PhCs is the existence of PBG, which prevents light propagation. The periodicity of the PhC can be disturbed to produce a defect mode inside the PBG allowing incident wave propagation within a narrow frequency range. The design of filters^7^, resonant cavities^8^, waveguides^9^, and nonlinear optical devices^10, 11^ could all benefit from using the defect modes.

Terahertz (THz) frequency spectrum has recently received a lot of attention due to its promising applications in biology and medicine^12^, telecommunications^13^, and spectroscopy^14^. Various THz functional devices, including THz sensors^15^, filters^16, 17^, and polarizers^18^ based on PhCs, are vital for these applications. Tuning the optical properties of PhCs offers new opportunities for developing technological and scientific applications. Tuning can be accomplished using dispersive materials such as metals^19^, liquid crystals^20–22^, and graphene^23, 24^. The dynamic control of permittivity is possible with these materials. However, they have drawbacks, such as remarkable absorption in metals, low tuning response in liquid crystals, and fabrication challenges in graphene nanolayers. Therefore, finding easily-prepared three-dimensional (3D) materials with tunable refractive indices is very important. Consequently, it is essential to find easily fabricated 3D materials with tunable refractive indices to realize dynamically tunable PhCs.

Bulk Dirac semimetals (BDSs) have also recently attracted considerable attention due to their ability in light manipulation^25–28^. Similar to graphene, these topological materials have linear dispersion relations in momentum space^26, 29, 30^. The refractive index of a BDS can be tuned by changing its Fermi energy via applying an external gate voltage^31^. At frequencies below and above the Fermi energy, these materials exhibit a metallic and a dielectric behavior, respectively.

Quasi-periodic PhCs (QPhCs) can be produced using the Thue-Morse and Fibonacci sequences^32, 33^. These structures have both periodic and random features, and their transmission spectra show isolated high-quality factor peaks, multiple PBGs, and periodic defects. The development of many optoelectronic devices, such as optical filters^34^, fibers^35^, wavelength division multiplexing^36^, sensors^37–43^, and laser diodes^44, 45^, which are exploited in diverse optical communication applications, is facilitated by all these properties. Moreover, due to the rise in the number of wavelengths sent and received in communication channels, as well as the compact form of these components, the use of tunable filters in wavelength distribution systems has recently attracted a lot of interest^46, 47^. For example, Belhaji et al. studied a tunable narrowband THz multichannel filter based on one-dimensional (1D) graphene-dielectric PhC by taking into account the impact of structural parameters such as incident angle of light, thickness, and refractive index of the dielectric layers as well as the chemical potential of graphene sheet^48^. Trabelsi et al. studied a tunable narrowband optical filter using superconductor-dielectric generalized Thue-Morse PhC^49^. Li et al*.* studied a 1D multiband graphene PhC filter in the THz region and investigated its tuning properties by changing the graphene chemical potential^50^.

In this study, we consider the third-order Fibonacci 1D PhC (F-1D PhC) structure containing BDS to design an optical filter. Using the new transfer matrix method, we theoretically analyzed the transmission spectrum of the structure as a function of the structural parameters. It is shown that the transmission spectrum of the structure behaves as a multichannel optical filter and represents a number of separate optical channels for both transverse electric (TE) and transverse magnetic (TM) polarizations. These optical channels are more sensitive to changes in the Fermi energy of BDS and the angle of incident light. Therefore, a dynamically tunable multichannel QPhC-based optical filter can be realized. Moreover, the effect of the periodic number of the Fibonacci structure is investigated, in which the number of channels is increased when the periodic number increases.

## Model and theory

**Figure 1:**
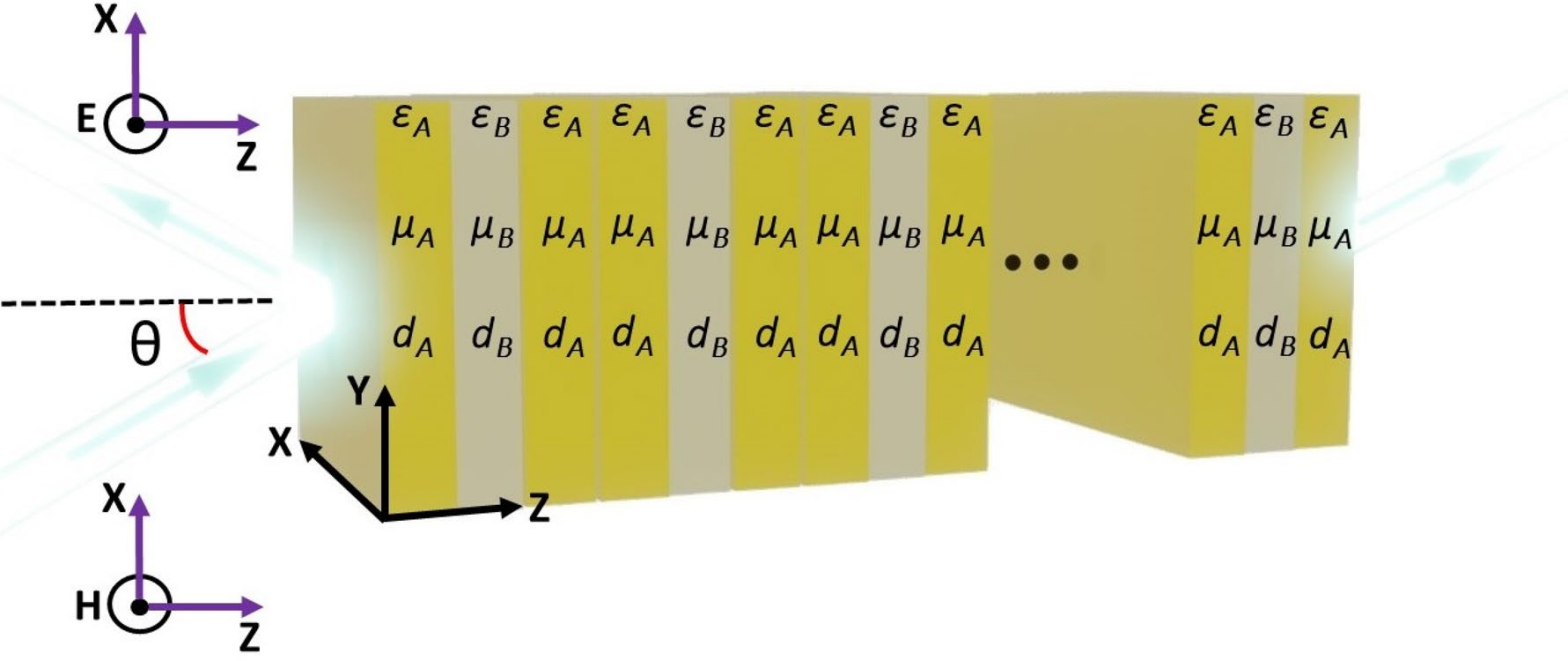
Schematic representation of a third-order F-1D PhC made up of BDS material and dielectric materials shown by alphabets $A$ and $B$, respectively.

In this section, we investigate the theoretical model describing the propagation of electromagnetic waves in a third-order F-1D PhC. The primary cell of the structure, as shown in Fig. 1, is ${F}_{3}=[ABA]$. Here alphabets $A$ and $B$ are used to represent BDS and dielectric layers of thickness, permeability and permittivity ${d}_{A}$, ${\mu }_{A}$, ${\varepsilon }_{A}$ and ${\varepsilon }_{B}$, ${\mu }_{B}$, ${d}_{B}$, respectively. The third-order F-1D PhC structure is made by stacking the ${F}_{3}$ unit cell for $N$ times, as shown in Fig. 1, where $N$ is the period number of the structure.

Based on the random phases approximation (RPA), the dielectric function of BDS material, ${\varepsilon }_{A}$, is defined as^51^:

1a $${\varepsilon }_{A}\left(\omega \right)={\varepsilon }_{b}+\frac{i{\sigma }_{DS}}{\omega {\varepsilon }_{0}}$$

where ${\varepsilon }_{b}$ is the background permittivity, ${\sigma }_{DS}$ is the optical conductivity of the $DS$ material with the following real and imaginary parts:

1b $$\mathrm{Re\,}{\sigma }_{DS}\left(\Omega \right)=\frac{{e}^{2}}{\hbar }\frac{\mathrm{g}{k}_{F}}{24\pi }G(\Omega /2)$$

1c $$\mathrm{Im }{\sigma }_{DS}\left(\Omega \right)=\frac{{e}^{2}}{\hbar }\frac{\mathrm{g}{k}_{F}}{24\pi }\left[\frac{4}{\Omega }\left(1+\frac{{\pi }^{2}}{3}{\left(\frac{T}{{E}_{F}}\right)}^{2}\right)+8\Omega {\int }_{0}^{{\varepsilon }_{c}}\left(\frac{G\left(\varepsilon \right)-G(\Omega /2)}{{\Omega }^{2}-4{\varepsilon }^{2}}\right)\varepsilon d\varepsilon \right]$$

where $G\left(E\right)=n\left(-E\right)-n(E)$, $n(E)$ is the Fermi distribution function, ${E}_{F}$ is the Fermi energy, ${k}_{F}={E}_{F}/\hbar {\nu }_{F}$ is the Fermi momentum, ${\nu }_{F}={10}^{6}$ m/s is the Fermi velocity, $\Omega =\hbar \omega /{E}_{F}$ is the normalized frequency, ${\varepsilon }_{c}={E}_{c}/{E}_{F}$, ${E}_{c}$ is the cut off energy above in which the dispersion relation becomes nonlinear, *e* is the charge of an electron, $\hbar$ is the reduced Planck constant, and *g* is the degeneracy factor.

The interaction between the incident electromagnetic wave and the proposed structure can be described using the new transfer matrix method, which is based on propagation and dynamic matrices^52, 53^. The transfer matrix of the entire structure is expressed as follows:

2 $${M}_{tot}={D}_{\mathrm{0,1}}{P}_{1}{D}_{\mathrm{1,2}}{P}_{2}\cdots {P}_{N-1}{D}_{N-1,N}{P}_{N}{D}_{N,sub}=\left(\begin{array}{cc}{m}_{11}& {m}_{12}\\ {m}_{21}& {m}_{22}\end{array}\right)$$

in which ${P}_{j}$ is the propagation matrix in the ${j}{th}$ layer with thickness ${d}_{j}$, and ${D}_{j,j+1}$ is the dynamic matrix that relates the electric fields between two layers $j$ and $j+1$, defined as:

3 $${P}_{j}\left(d\right)=\left(\begin{array}{cc}{e}^{-i{k}_{z,j}}& 0\\ 0& {e}^{i{k}_{z,j}}\end{array}\right)$$

4 $${D}_{j,j+1}=\frac{1}{2}\left(\begin{array}{cc}1+\frac{{k}_{z,j+1}}{{k}_{z,j}}& 1-\frac{{k}_{z,j+1}}{{k}_{z,j}}\\ 1-\frac{{k}_{z,j+1}}{{k}_{z,j}}& 1+\frac{{k}_{z,j+1}}{{k}_{z,j}}\end{array}\right)$$

where ${k}_{z,j}$ is the z-component of the wave vector in ${j}{th}$ layer, which is equal to ${k}_{z,j}={k}_{z,j}$ for TE polarization and ${k}_{z,j}={k}_{z,j}/{\varepsilon }_{j}$ for TM polarization and is defined as:

5 $$k_{{z,j}} = \sqrt {\left( {\frac{\omega }{c}} \right)^{2} \varepsilon _{j} - k_{x}^{2} }$$

in which $\omega$ is the angular frequency, $c$ is the speed of light, and ${\varepsilon }_{j}$ is the permittivity of the ${j}^{th}$ layer. The transmission spectrum of the structure is written as:

6 $$T={\left|\frac{1}{{m}_{11}}\right|}^{2}$$

## Proposal for fabrication and testing

As per the understanding of the authors toward the fabrication part of the proposed third-order F-1D PhC made up of topological Dirac semimetal ${\mathrm{Na}}_{3}\mathrm{Bi}$ and ${\mathrm{SiO}}_{2}$ or $\mathrm{Si}$ layers, the proposed structure can be fabricated by using molecular beam epitaxy fabrication technique^54, 55^. Other conventional fabrication techniques may also be useful for the development of the structure. After fabricating the third-order F-1D PhC we can examine the performance of the structure by using terahertz (THz) time domain spectroscopy (TDS) for testing the multichannel filtering properties of the proposed PhC. The THz TDS system comprises of five basic parts. The first part of THz TDS system is ultra-fast THz femtosecond laser which is used to launch laser light into the proposed F-1D PhC through splitter which splits the incident laser light into pump and probe beams. The pump beam is fall on the second part called as THz emitter unit for creating THz pulses ranges from 0.1 to 11 THz. The synchronized THz radiation is then allowed to inject into third part of the system which is F-1D PhC via parabolic mirrors and inlet valve. The fourth part is THz InGaAs-based antenna for detection of electric field intensity of the THz radiation coming out from F-1D PhC though outlet valve. Finally, delay unit as a fifth part of the THz TDS system is used for offsetting the pump and probe pulses to allow the repetitive sampling of THz signal for temporal analysis.

## Results and discussion

In this study, we numerically investigate the ability of the proposed third-order F-1D PhC structure to produce a multichannel optical filter by optimizing its structural and geometrical parameters. Then, the tuning properties of the optical channels are examined by varying the Fermi energy of the BDS layer at temperature $T=70$ K. To explain why this temperature was chosen, it is necessary to note that the transmission peak of optical channels decreases for temperatures above $70$ K and shows a slight increase at temperatures below it. Moreover, we investigate the effect of the light incident angle and number of periodicity on optical channels. For this purpose, the transmission spectrum of the structure will be separately analyzed using the new transfer matrix method for both TE and TM polarizations in the following two subsections.

### TE polarization

**Figure 2:**
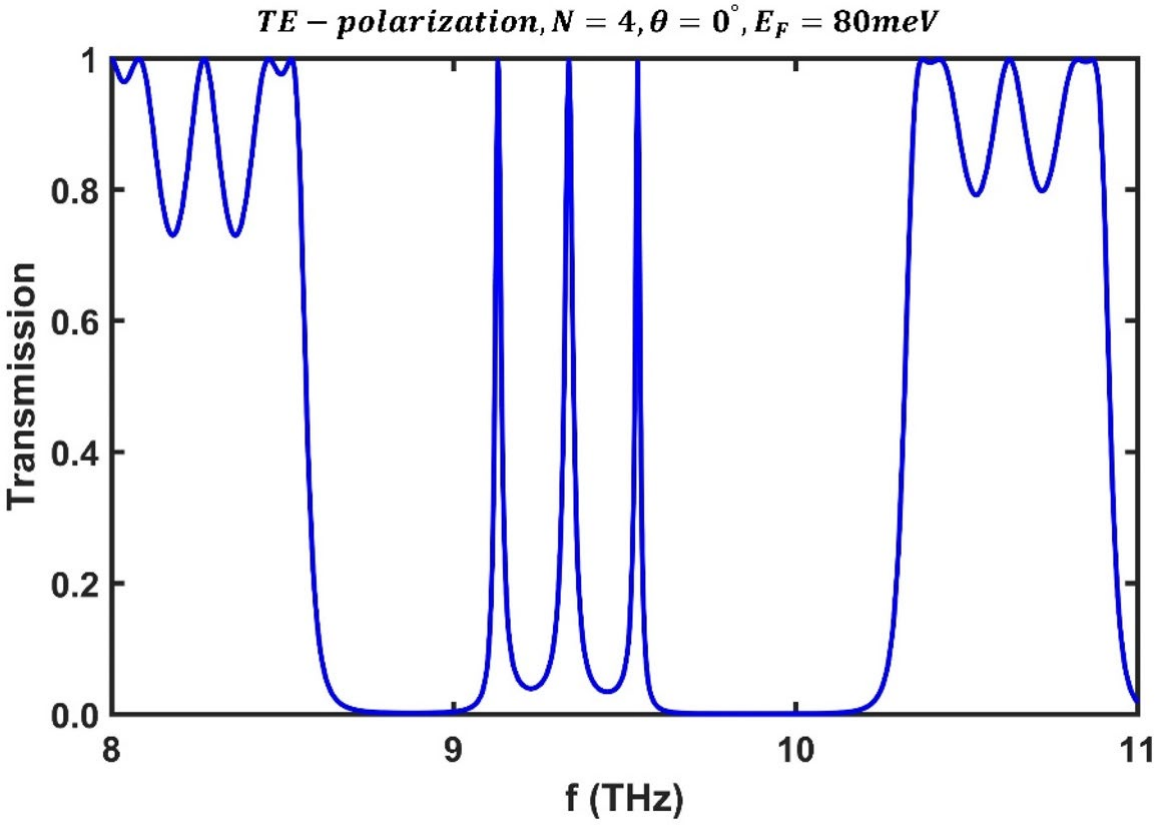
Transmission spectrum of the third-order F-1D PhC under normal incidence for TE polarization state corresponding to Fermi energy ${E}_{F}=80$ meV and period number $N=4$.

First, we examined our structure under TE polarization. The BDS layer is considered to be ${\mathrm{Na}}_{3}\mathrm{Bi}$ or ${\mathrm{Cd}}_{3}{\mathrm{As}}_{2}$ with ${\varepsilon }_{b}=13$ and $g=4$^56^. The frequency-dependent dielectric permittivity of the layer $A$ (i.e. BDS layer) is calculated using Eq. (1a)–(1c) and its permeability and thickness were selected as ${\mu }_{A}=3$ and ${d}_{A}=6$ μm. The layer $B$ is ${\mathrm{SiO}}_{2}$ with thickness ${d}_{B}=3$ μm, dielectric constant ${\varepsilon }_{B}=2.25$, and permeability ${\mu }_{B}=1$^57^. Using the data provided, under the perpendicular irradiation of the incident light, the transmission spectrum of the proposed structure was calculated at Fermi energy ${E}_{F}=80$ meV for $N=4$ periods, as shown in Fig. 2.

As can be seen from Fig. 2, this structure has three optical channels for the zero-incident angle and can operate as a triple optical filter. It should be noted that the appearance of multiple isolated peaks in the transmission spectrum is due to the structural properties of the F-1D PhC, not due to defects. Our findings show that higher-order F-1D PhCs lack these filtering characteristics. Numerical calculations show that the frequencies of the channels are ${f}_{1}=9.13$ THz, ${f}_{2}=9.33$ THz, and ${f}_{3}=9.538$ THz, and their corresponding peak values are $0.9963$, $0.9963$, and $0.9944$, respectively. Following that, we examined in detail the effect of Fermi energy (${E}_{F}$), radiation angle ($\theta$), and period number ($N$) on a constructed multichannel optical filter.

**Figure 3:**
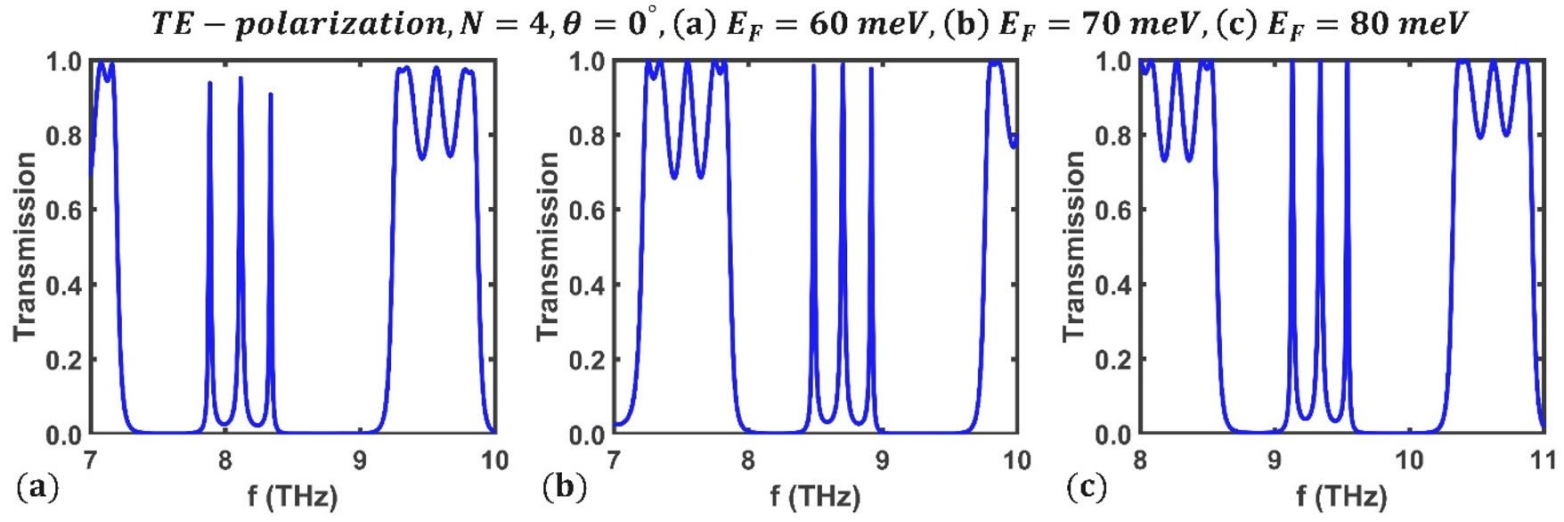
Transmission spectrum of the third-order F-1D PhC under normal incidence condition for TE polarization corresponding to different Fermi energies, (**a**) ${E}_{F}=60$ meV, (**b**) ${E}_{F}=70$ meV, and (**c**) ${E}_{F}=80$ meV.

First, we studied the effect of Fermi energy on the transmission spectrum of the designed multichannel optical filter. Figure 3 shows the obtained results for TE polarization under perpendicular radiation at three different values of Fermi energy, ${E}_{F}=60, 70, 80$ meV, and $N=4$ periods. It is observed that the change in Fermi energy leads to a change in the frequency and peak value of each optical channel. Numerical results show that the frequencies of the channels at ${E}_{F}=60$ meV are ${f}_{1}=7.885$ THz, ${f}_{2}=8.114$ THz and ${f}_{3}=8.336$ THz, and their peak values are $0.9384$, $0.9516$, and $0.9085$, respectively. The frequencies and peak values of the optical channels at ${E}_{F}=80$ meV are reported in Fig. 2. It is clear that optical channels' frequency shifts to higher values, and their peak values increases. These results provide the design of a tunable multichannel optical filter by changing the BDS's Fermi energy. It should be noted that in practice, applying a gate voltage^58–61^ or doping an alkaline surface^29, 51^ can dynamically change the Fermi energy and, as a result, the refractive index of the BDS.

**Figure 4:**
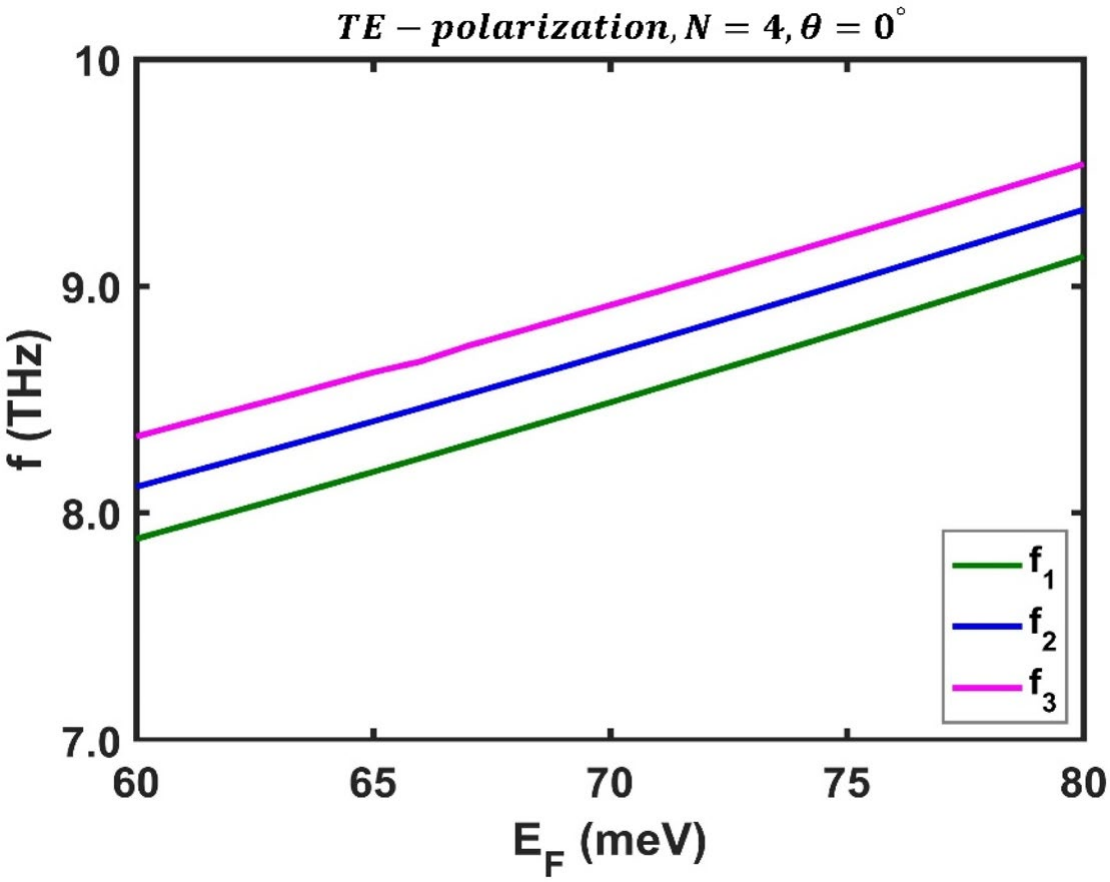
Variation of central frequency of all three the optical channels' in terms of Fermi energy for TE polarization state under normal incidence in a third-order F-1D PhC structure with $N=4$. The central frequencies of all three optical channels shown in green, blue and magenta colors and ranges from ${(f}_{1}, {f}_{2}, {f}_{3})=(7.885, 8.114, 8.336)$ THz at ${E}_{F}=60$ meV to ${(f}_{1}, {f}_{2}, {f}_{3})=(9.13, 9.33, 9.538)$ THz at ${E}_{F}=80$ meV.

Then, for a comprehensive investigation, the frequency of the optical channels was plotted as a function of Fermi energy under perpendicular irradiation for TE polarization, as shown in Fig. 4. It is evident that as the Fermi energy increases, all the optical channels' frequencies increase linearly. These findings demonstrate that the Fermi energy has a remarkable effect on controlling the frequency of the optical channels and shows the ability of the desired structure as a tunable multichannel optical filter. The numerical results show that the frequency of the optical channels vary from ${f}_{1}=7.885$ THz, ${f}_{2}=8.114$ THz and ${f}_{3}=8.336$ THz at ${E}_{F}=60$ meV to ${f}_{1}=9.13$ THz, ${f}_{2}=9.33$ THz, and ${f}_{3}=9.538$ THz at ${E}_{F}=80$ meV.

**Figure 5:**
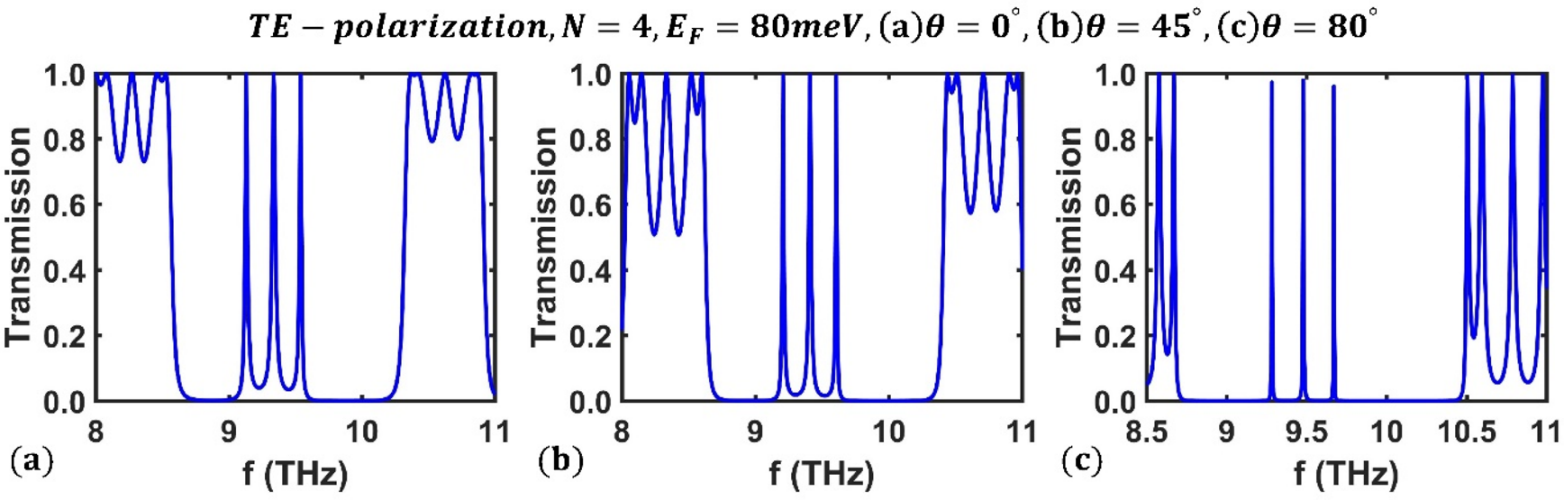
Transmission spectrum of the third-order F-1D PhC for TE polarization state corresponding to fixed values of Fermi energy and period number as ${E}_{F}=80$ meV and $N=4$, respectively, at incident angles (**a**) ${\theta=0}^{^\circ }$, (**b**) $\theta ={45}^{^\circ }$, and (**c**) $\theta ={80}^{^\circ }$.

Next, we examine the effect of the light incident angle on the optical channels of the designed filter. As an example, we took the Fermi energy of BDS material as ${E}_{F}=80$ meV to explore the effect of the incident angle on the transmission spectrum of the third-order F-1D PhC with $N=4$ periods for TE polarization. The obtained results at three incident angles ${\theta=0}^{^\circ }$, ${\theta =45}^{^\circ }$, and $\theta ={80}^{^\circ }$, are shown in Fig. 5. As can be seen, an increase in radiation angle causes an upward shift in the frequency of optical channels.

**Figure 6:**
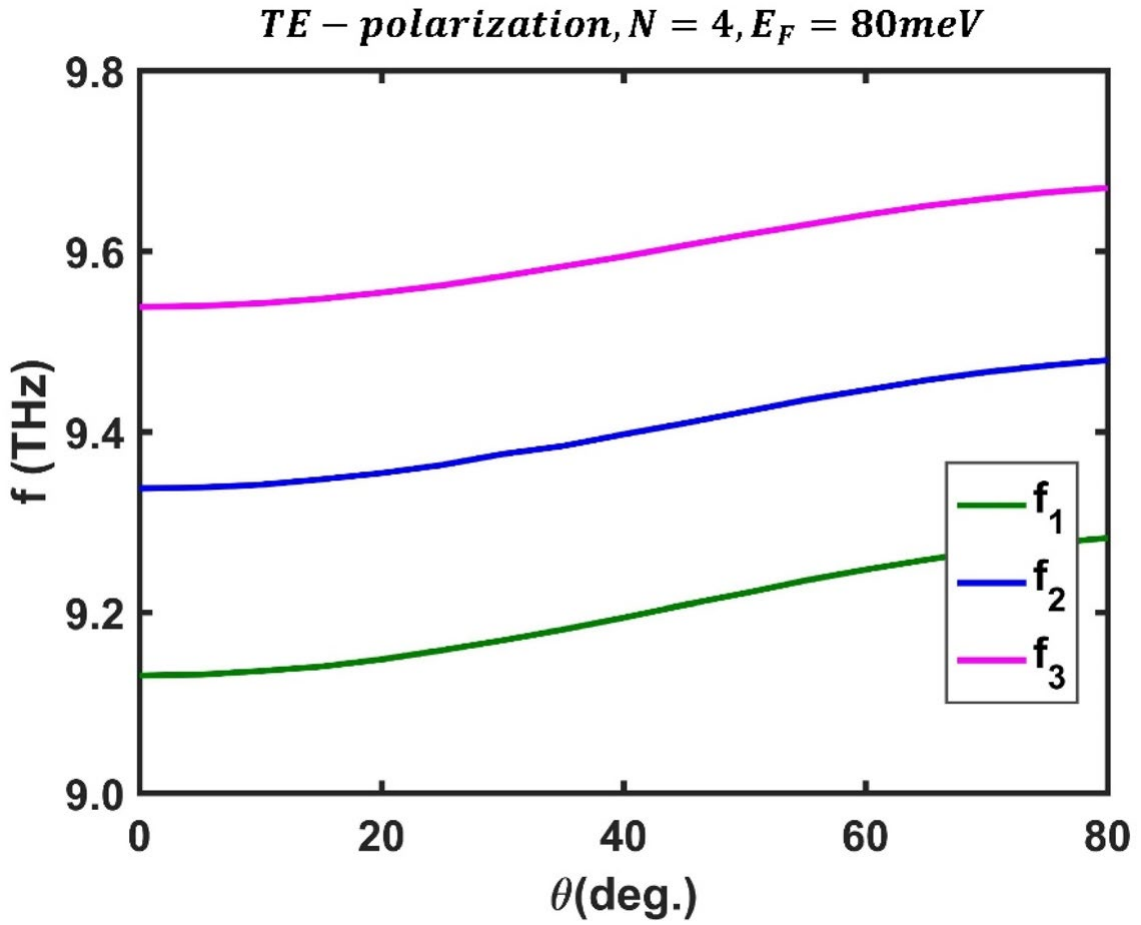
Dependence of central frequency of all three optical channels on the incident angle for TE polarization state at fixed values of ${E}_{F}=80$ meV and $N=4$ in third-order F-1D PhC. The central frequencies of all three optical channels shown in green, blue and magenta colors and changes from ${(f}_{1}, {f}_{2}, {f}_{3})=(9.13, 9.337, 9.53)$ THz at $\theta =0$ to ${(f}_{1}, {f}_{2}, {f}_{3})=(9.282, 9.479, 9.67)$ THz at $\theta ={80}^{^\circ }$.

At an energy of ${E}_{F}=80$ meV, Fig. 6 shows the variation in optical channel frequencies as a function of incident angle, where the frequencies of the optical channels at $\theta ={0}^{^\circ }$ are ${f}_{1}=9.13$ THz, ${f}_{2}=9.337$ THz, and ${f}_{3}=9.53$ THz; they reach values of ${f}_{1}=9.282$ THz, ${f}_{2}=9.479$ THz, and ${f}_{3}=9.67$ THz at an incident angle of $\theta ={80}^{^\circ }$. Additionally, within the same range of incident angle, the peak value of the first optical channel ranges from $0.9963$ to $0.9728$, the second from $0.9971$ to $0.9792$, and the third from $0.9944$ to $0.9604$. Therefore, the frequencies of the optical channels changes in response to variations in the radiation angle of the incident electromagnetic wave, implying that the designed filter can be tuned by changing the light incident angle.

**Figure 7:**
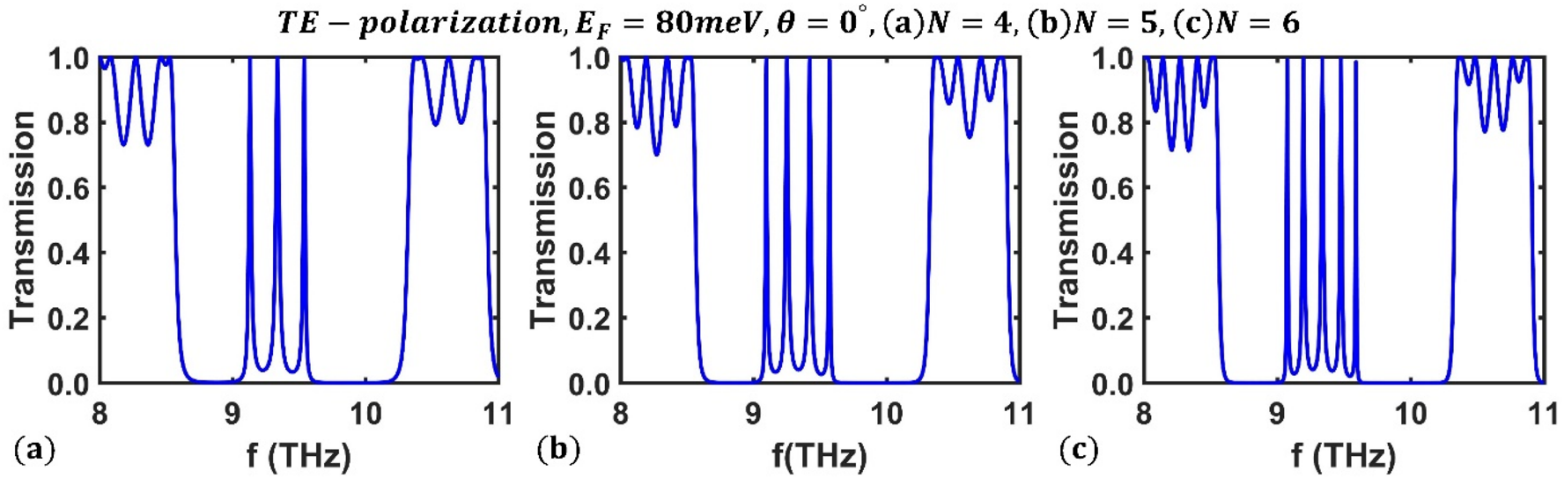
Transmission spectrum of the third-order F-1D PhC for TE polarization state under normal incidence corresponding to Fermi energy ${E}_{F}=80$ meV and period numbers (**a**) $N=4$ periods, (**b**) $N=5$ periods, and (**c**) $N=6$.

Finally, for a given Fermi energy, we looked into how the periodic number ($N$) affects the optical channels of the designed filter. Results for a Fermi energy of ${E}_{F}=80$ meV and $N=4$, $N=5$, and $N=6$ periods are shown in Fig. 7 for TE polarization under perpendicular radiation. As can be seen, the transmission spectrum of the structure exhibits $N-1$ optical channels for each value of $N$, and the number of channels rises as the periodic number increases. Therefore, the appropriate multichannel optical filter can be obtained by varying the structure's periodic number.

### TM polarization

**Figure 8:**
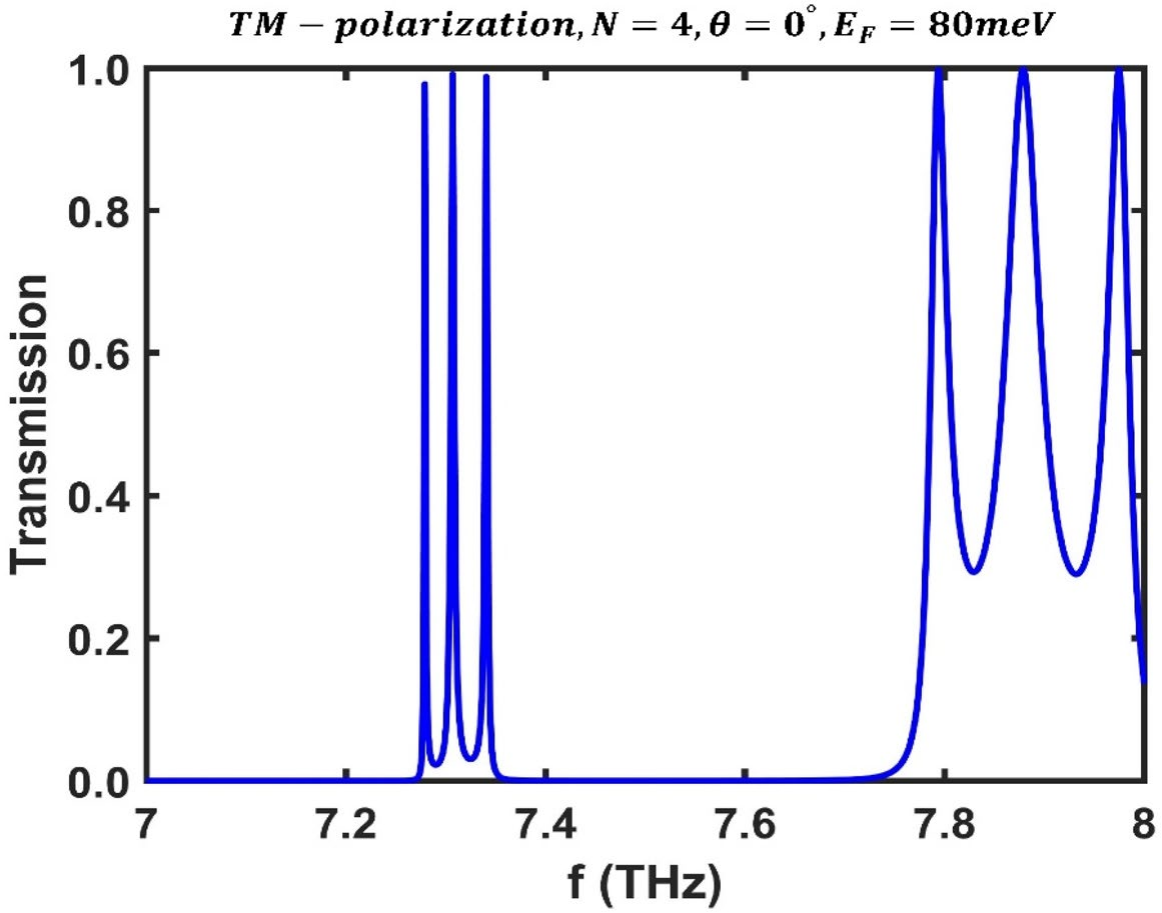
Transmission spectrum of the third-order F-1D PhC for TM polarization state under normal incidence corresponding to Fermi energy ${E}_{F}=80$ meV and period number $N=4$.

In this section, we investigate the transmission spectrum of the third-order F-1D PhC structure for TM polarization and optimize its geometrical and physical parameters to produce a multichannel optical filter. In this case, a different dielectric material was employed, but the same BDS material with a thickness of ${d}_{A}=7$ μm was used. Dielectric $B$ is $Si,$ and the permeability, thickness, and dielectric constant are ${\mu }_{B}=1$, ${d}_{B}=2$ μm, and ${\varepsilon }_{B}=11.9$, respectively^58^. The results show that the transmission spectrum of the proposed structure, when exposed to incident light at a perpendicular angle, exhibits three optical channels for a given Fermi energy ${E}_{F}=80$ meV and $N=4$ periods, as shown in Fig. 8. This structure, a type of multichannel filter, has the capability to act as a triple optical filter.

Numerical results show that the optical channels have frequencies of ${f}_{1}=7.279$ THz, ${f}_{2}=7.307$ THz, and ${f}_{3}=7.341$ THz, with peak values of $0.9777$, $0.9915$, and $0.988$, respectively.

**Figure 9:**
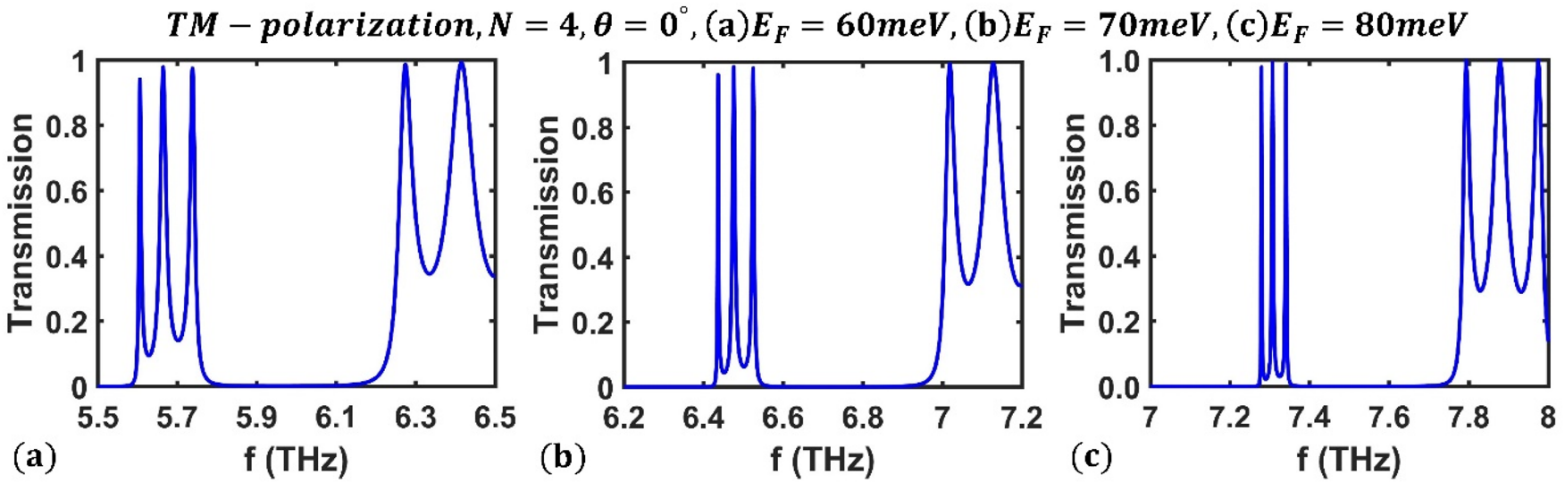
The transmission spectrum of the third-order F-1D PhC for TM polarization state under normal incidence corresponding to period numbers $N=4$ and Fermi energies, (**a**) ${E}_{F}=60$ meV, (**b**) ${E}_{F}=70$ meV, and (**c**) ${E}_{F}=80$ meV.

**Figure 10:**
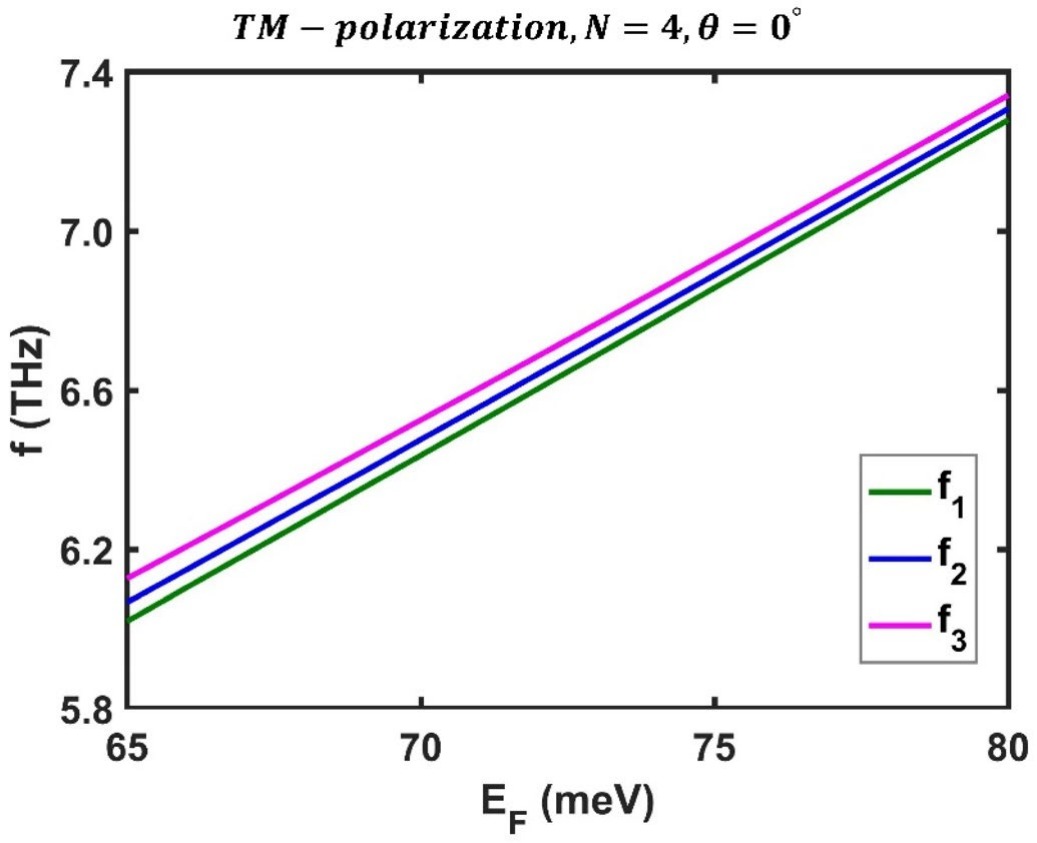
Variation of central frequency of all three optical channels with respect to Fermi energy for TM polarization state under normal incidence in the third-order F-1D PhC structure corresponding to period number $N=4$. The central frequencies of all three optical channels are shown in green, blue and magenta and changes from ${(f}_{1}, {f}_{2}, {f}_{3})=(5.606, 5.664, 5.738)$ THz at ${E}_{F}=60$ meV to ${(f}_{1}, {f}_{2}, {f}_{3})=(7.279, 7.307, 7.341)$ THz at ${E}_{F}=80$ meV.

Then, similar to the previous subsection, we investigated the effect of Fermi energy (${E}_{F}$), incident angle ($\theta$), and periodic number ($N$) on optical filters appearing in the transmission spectrum of the F-1D PhC structure for TM polarization. First, we examine the effect of Fermi energy under perpendicular radiation for $N=4$ periods. Figure 9 shows the transmission spectrum of the proposed structure for TM polarization at three distinct Fermi energies $60$ meV, $70$ meV, and $80\mathrm{ meV}$. It is evident that, as the Fermi energy rises, the frequency of optical channels shifts upward and range from $5.606$, $5.664$, and 5 $.738$ THz at ${E}_{F}=60$ meV to $7.279$, $7.307$, and $7.341$ THz at ${E}_{F}=80$ meV. These numerical findings show that the amounts of tuning for optical channels are $1.673$ THz, $1.643$ THz, and $1.603$ THz. Additionally, within the same Fermi energy range, the optical channels' peak values range from $0.9411$, $0.9777$, and $0.9739$ to $0.9777$, $0.9915$, and $0.988$. These results show that the proposed multichannel optical filter can be tuned by adjusting the Fermi energy of BDS material. Figure 10 shows how the Fermi energy affects the frequency of optical channels in the F-1D PhC structure for TM polarization under perpendicular radiation.

**Figure 11:**
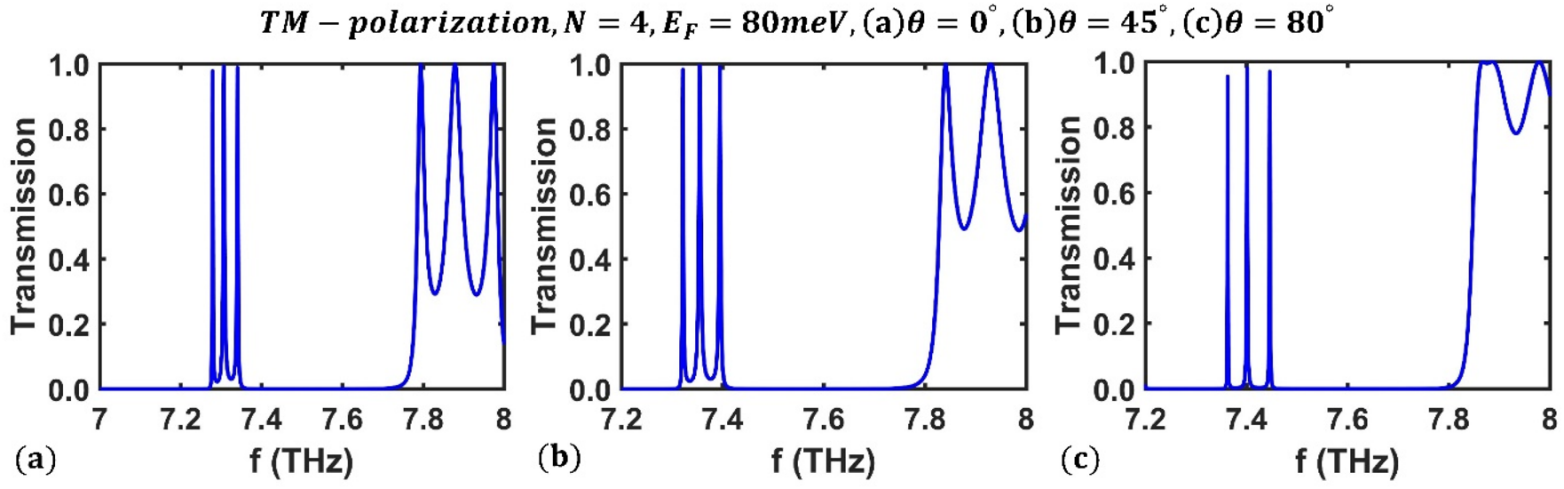
The transmission spectrum of the third-order F-1D PhC for TM polarization state corresponding to fixed values of Fermi energy and period number as ${E}_{F}=80$ meV and $N=4$, respectively, under different light incident angles (**a**) $\theta={0}^{^\circ }$, (**b**) $\theta ={45}^{^\circ }$ and (**c**) $\theta ={80}^{^\circ }$.

Next, we look at how the incidence angle of the designed filter affects its optical channels. The transmission spectrum of the designed filter based on the third-order F-1D PhC for TM polarization at three different values of incident angles, $\theta ={0}^{^\circ }$, $\theta ={45}^{^\circ }$ and $\theta ={80}^{^\circ }$, is shown in Fig. 11, assuming the Fermi energy of ${E}_{F}=80$ meV and $N=4$ periods.

**Figure 12:**
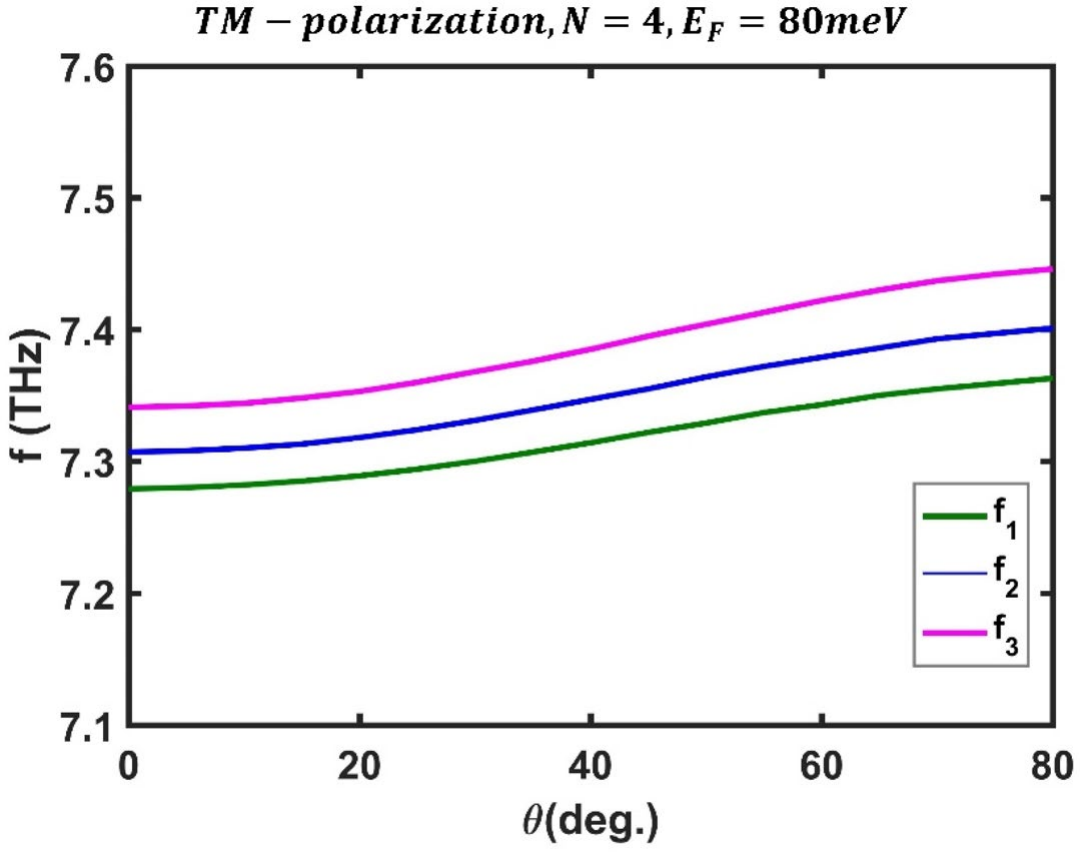
Dependence of central frequency of all three optical channels on the light incident angle for TM polarization state in third-order F-1D PhC structure at fixed values of ${E}_{F}=80$ meV and $N=4$. The central frequencies of all three optical channels are shown in green, blue and magenta colors and changes from ${(f}_{1}, {f}_{2}, {f}_{3})=(7.279, 7.307, 7.341)$ THz at $\theta =0$ to ${(f}_{1}, {f}_{2}, {f}_{3})=(7.363, 7.401, 7.446)$ THz at $\theta ={80}^{^\circ }$.

As can be seen, increasing the radiation angle shifts the frequency of the optical channels toward high frequencies. Figure 12 shows the frequency variation of optical channels versus the incident angle for a Fermi energy of ${E}_{F}=80$ meV and $N=4$ periods. The obtained results show that when the incident angle changes from $\theta ={0}^{^\circ }$ to $\theta ={80}^{^\circ }$, the frequencies of the optical channels range from $7.279$ THz, $7.307$ THz, and $7.341$ THz to $7.363$ THz, $7.401$ THz, and $7.446$ THz. The corresponding peak values of the optical channels are $0.9777$, $0.9915$, and $0.988$ at $\theta ={0}^{^\circ }$, and they reach the values $0.9543$, $0.9798$, and $0.9694$ when the light incident angle is $\theta ={80}^{^\circ }$. Therefore, changes in the radiation angle of the electromagnetic wave are crucial in tuning the frequency of optical channels.

**Figure 13:**
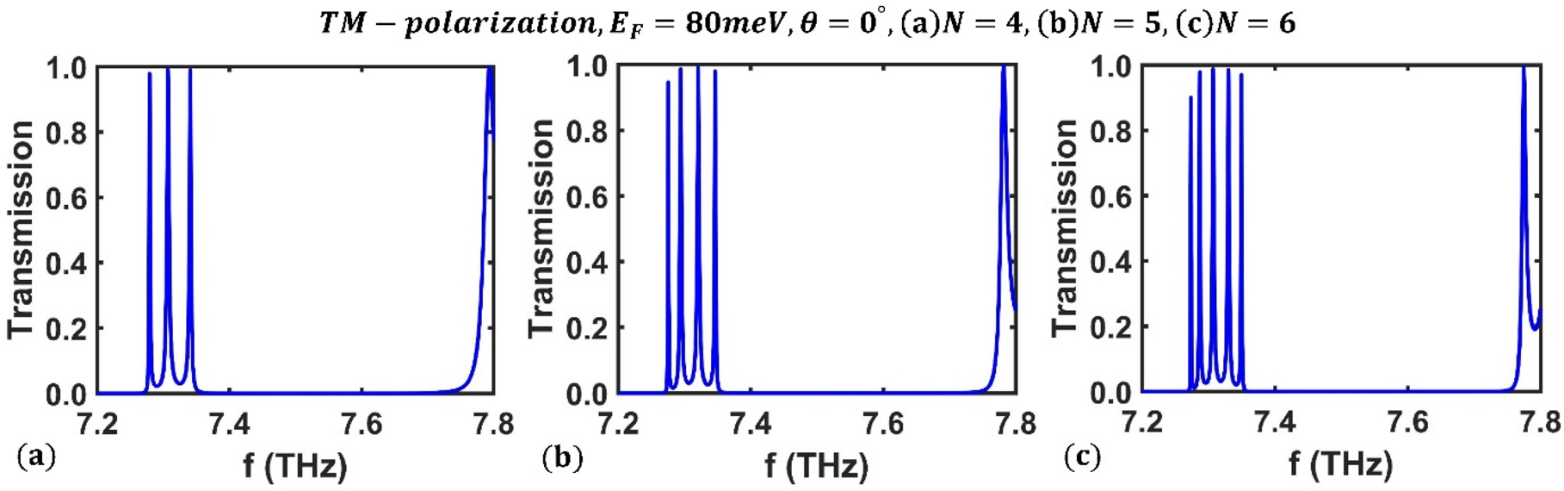
The transmission spectrum of the third-order F-1D PhC structure for TM polarization state under normal incidence corresponding to Fermi energy ${E}_{F}=80$ meV and different period numbers (**a**) $N=4$, (**b**) $N=5$ and (**c**) $N=6$.

Finally, we explored how periodicity ($N$) affects the transmission spectrum of the third-order F-1D PhC structure for TM polarization under incident light that is radiated perpendicularly at a fixed value of Fermi energy. The numerical results for three different values of $=4$, $N=5$, and $N=6$ periods are shown in Fig. 13 for a particular Fermi energy of ${E}_{F}=80$ meV. This figure shows that as the periodic number ($N$) increases, so does the number of optical channels. It is evident that given a periodicity of N, the transmission spectrum of the F-1D PhC shows $N-1$ optical channels. Therefore, the appropriate multichannel optical filter can be achieved by changing the periodic number of the structure.

## Conclusion

We proposed a third-order F-1D PhC structure composed of BDS material and a dielectric layer and investigated its transmission spectrum using new transfer matrix method in the THz frequency region for both TE and TM polarizations. Results demonstrated that a few optical channels, or multichannel optical filters, appeared in the transmission spectrum of the structure for both polarizations. The tuning properties of the optical channels were then theoretically investigated by varying the light incident angle and the BDS's Fermi energy. The numerical results show that these parameters significantly affect the frequency of the optical channels. Next, the influence of the Fibonacci structure's periodic number on optical channels was explored. The results demonstrate that increasing the periodicity can increase the number of optical channels. These results may be useful in designing integrated photonic devices based on F-1D PhC.

## Data Availability

The datasets used and/or analyzed during the current study available from the corresponding author on reasonable request.
